# Identification of palliative care needs and prognostic factors of survival for tailoring appropriate interventions in advanced oncological, renal, and pulmonary diseases: results from a prospective observational study in an Italian population

**DOI:** 10.1186/s12904-026-02257-z

**Published:** 2026-08-05

**Authors:** Emanuela Scarpi, Vanessa Valenti, Monia Dall’Agata, Ilaria Bassi, Lucia Angelini, Andrea Carlini, Paola Cravero, Angelo Delmonte, Gaetano La Manna, Giacomo Magnoni, Martina Marchello, Giovanni Mosconi, Oriana Nanni, Stefano Nava, Margherita Currà, Maria Caterina Pallotti, Ilario Giovanni Rapposelli, Marianna Ricci, Anna Scrivo, Alessandra Spazzoli, Danila Valenti, Loretta Zambianchi, Augusto Caraceni, Marco Maltoni, Romina Rossi

**Affiliations:** 1 Unit of Biostatistics and Clinical Trials, IRCCS Istituto Romagnolo per lo Studio dei Tumori (IRST) “Dino Amadori”, Via P. Maroncelli 40, Meldola, FC 47014 Italy; 2 Palliative Care Unit, IRCCS Istituto Romagnolo per lo Studio dei Tumori (IRST) “Dino Amadori”, Meldola, Italy; 3 Respiratory and Critical Care Unit, IRCCS Azienda Ospedaliero Universitaria di Bologna, Bologna, Italy; 4 Department of Medical and Surgical Sciences (DIMEC), Alma Mater Studiorum - University of Bologna, Bologna, Italy; 5 Medical Oncology Unit, Department of Medical and Surgical Sciences (DIMEC), Alma Mater Studiorum - University of Bologna, Bologna, Italy; 6 Department of Medical Oncology, IRCCS Istituto Romagnolo per lo Studio dei Tumori (IRST) “Dino Amadori”, Meldola, Italy; 7 Nephrology, Dialysis and Kidney Transplant Unit, IRCCS Azienda Ospedaliero Universitaria di Bologna, Bologna, Italy; 8 Nephrology and Dialysis Unit, AUSL della Romagna, “Morgagni-Pierantoni” Hospital, Forlì, Italy; 9 Unità Operativa Complessa Rete delle Cure Palliative, AUSL di Bologna (Dipartimento dell’Integrazione), Bologna, Italy; 10 Department of Palliative Care Ravenna, AUSL Romagna, Ravenna, Italy; 11 Palliative Care, Pain Therapy and Rehabilitation Unit, Fondazione IRCCS Istituto Nazionale dei Tumori, Milan, Italy; 12 Radiation Oncology, Department of Medical and Surgical Sciences (DIMEC), Alma Mater Studiorum - University of Bologna, Bologna, Italy

**Keywords:** Early outpatient palliative care, Chronic illness, Palliative care integration, Palliative care identification tool, Palliative care need, Advanced cancer and patients with non-oncological diseases

## Abstract

**Background:**

The increasing prevalence of chronic diseases makes identifying patients in need of palliative care essential for public health systems. Several tools have been developed to identify patients with palliative care needs and limited life expectancy. This study aimed to evaluate the performance of the NECPAL and PaP tools in patients with advanced chronic diseases and to explore their potential role in identifying palliative care needs and prognostic assessment.

**Methods:**

Prospective, observational, multicenter study using the Necesidades Paliativas tool (NECPAL CCOMS-ICO version 1.0), Palliative Prognostic score (PaP score), and functional and symptom assessment tools (Palliative Performance Scale and Edmonton Symptom Assessment System), assessed monthly or every two months for up to 12 months.

Information on healthcare utilization and end-of-life care was also collected. Sensitivity, specificity, positive/negative predictive value and accuracy were calculated. The study included three groups of patients with advanced chronic diseases: cancer, pulmonary diseases, and renal diseases. The study was reported following the STROBE guidelines.

**Results:**

One hundred and ninety-three patients were enrolled: 102 with advanced cancer, 50 with chronic respiratory failure, and 41 with chronic renal failure. The median age was 75 years and 63.7% were male. The overall survival rate was 90% at 30 days and 65% at 12 months. At 30 days, sensitivity and specificity were 100% and 88% for the PaP score, and 89% and 39% for NECPAL. At 12 months, sensitivity and specificity were 20% and 89% for the PaP score, and 88% and 41% for NECPAL. Prognostic accuracy was higher for the PaP score at 30 days (91%) and higher for the NECPAL tool at 12 months (81%). Patients with cancer had more frequent hospitalizations in acute units, while the people living with pulmonary disease had more total hospitalization days.

**Conclusions:**

The findings suggest that the two instruments may provide complementary information, although the potential benefit of a combined strategy was not directly evaluated in this study.

**Supplementary Information:**

The online version contains supplementary material available at https://doi.org/10.1186/s12904-026-02257-z.

## Background

Caring for patients with advanced chronic conditions represents a major challenge for healthcare systems worldwide. Over 56.8 million people are estimated to require palliative care each year, including 31.1 million before and 25.7 million near the end of life, yet only 14% receive it, mostly in high-income settings [1]. Population ageing and the rising burden of non-communicable diseases are expected to nearly double the number of deaths by 2060 [2], contributing to a substantial increase in serious health-related suffering requiring palliative care. It is estimated that by 2060, 48 million people will die experiencing such suffering, compared with 26 million in 2016 [3].

For these reasons, palliative care for patients with advanced chronic diseases and terminal illnesses, including cancer, heart failure, renal failure, and respiratory failure [4], is increasingly recognized as a cornerstone of global health. International and national frameworks support its integration across disease trajectories [5–7]. Growing evidence shows that timely palliative care improves quality of life [8–11], symptom control, and satisfaction for patients and caregivers, while reducing aggressive end-of-life care and healthcare utilization [12]. Although the earliest evidence on early integrated palliative care derives mainly from oncology populations, this approach has progressively been extended to patients with advanced non-oncological chronic diseases [13, 14]. However, its implementation requires timely identification of patients, assessment of needs, prognostication, and care planning. A major barrier to early integration is the lack of clinician awareness and difficulty in applying criteria to identify patients in need [15].

Several tools have been developed for identifying patients with palliative care needs, assessing complexity, and supporting prognostication, but their routine use remains limited [16–18].

Hui et al. identified new areas of research where new tools and/or prognostic factors, or a combination of both, were proposed to enhance prognostic processes [19]. Moreover, some instruments originally designed for prognostic purposes have also been repurposed as tools for identifying palliative care needs, and vice versa, highlighting conceptual overlap in clinical practice.

In this context, we used two validated instruments: the Necesidades Paliativas (NECPAL) tool and the Palliative Prognostic (PaP) score. NECPAL is primarily used to identify patients with palliative care needs [20], while PaP score provides short-term prognostic stratification, estimating 30-day survival [21–23]. Both tools have been validated in independent studies and in the Italian context. NECPAL has also been proposed for prognostic purposes, particularly for longer-term outcomes [24], whereas the PaP score was originally developed as a prognostic tool in advanced chronic diseases and oncologic population. Because NECPAL and PaP were developed for different purposes, evaluating their performance within the same population may provide useful information regarding their potential roles in identifying palliative care needs and prognostic assessment. If future studies confirm the value of integrating information from both instruments, such an approach may facilitate pathways including needs assessment, advance care planning, and case management within chronic disease management programs. Timely palliative care has been associated with reduced suffering, improved outcomes, and lower healthcare utilization and costs.

This study aims to evaluate the performance of NECPAL and PaP score in patients with advanced chronic diseases and to explore their potential role in patient identification and prognostic assessment.

## Methods

This study was conducted and reported in accordance with the STROBE guidelines for observational studies [25]. This prospective, observational, multicenter study involved patients with advanced chronic diseases, including cancer, chronic pulmonary diseases, and renal failure [26]. Eligibility required patients aged ≥ 18 years with an estimated life expectancy greater than two months, as assessed by the treating physician based on clinical judgement and disease trajectory, and at least one of the following conditions: (1) inoperable, locally advanced, or metastatic non-small cell lung cancer, gastric cancer, or pancreatic adenocarcinoma diagnosed within the last eight weeks; (2) chronic obstructive pulmonary disease (COPD) or idiopathic pulmonary fibrosis (IPF) with disease-specific indicators of advanced disease. COPD-specific criteria included severe airflow limitation, oxygen therapy dependence, previous hospitalization for exacerbation, relevant comorbidities, weight loss/cachexia, reduced functional autonomy, or increased dependence. IPF-specific criteria included oxygen therapy dependence, radiological and/or histological evidence of advanced fibrosis, reduced functional autonomy, or increased dependence; or (3) advanced chronic renal failure with characteristics including age over 75 and severe comorbidities. Specific cancer disease-related inclusion criteria were selected based on the Temel papers used by our group in previous studies [9, 11, 27]. Regarding the non-oncologic conditions included in the study, the disease-related inclusion criteria were defined according to a shared document developed by palliative care and intensive care societies on end-stage organ failure, which also set the age thresholds for pulmonary (> 70 years) and renal (> 75 years) populations, reflecting the distinct clinical trajectories and frailty profiles of these conditions [28]. Exclusion criteria included patients who had previously received palliative care or been admitted to hospice.

Patients were assessed at baseline and then monthly or bi-monthly for up to 12 months. Additional assessments were made if the patient’s condition worsened or if there was a change in care setting. Data collected included demographic information, PaP score [21, 22], NECPAL CCOMS-ICO version 1.0 tool [20], Edmonton Symptom Assessment System (ESAS) [29], Palliative Performance Scale (PPS) [30], and Eastern Cooperative Oncology Group (ECOG) performance status scale [31]. For people living with pulmonary disease, dyspnea and cough were also evaluated using the Modified Borg Scale (MBS) [32] and Numerical Rating Scale (NRS), respectively. At the end of the study, data on the use of health services and end-of-life care (including medical prescriptions, referral to hospice, inclusion in the local palliative care network, hospital admissions, emergency department visits, Intensive Care Unit (ICU) admissions, and date and location of death) were also collected as exploratory descriptive outcomes to provide contextual information on care trajectories.

The PaP score is a prognostic tool based on six factors: Karnofsky Performance Status, clinical survival prediction, anorexia, dyspnea, white blood cell count, and lymphocyte percentage. It predicts 30-day survival by categorizing patients into three risk groups.

The NECPAL tool uses the Surprise Question (SQ) [33, 34], asking clinicians if they would be surprised if the patient died in the next 12 months, along with additional clinical criteria. Patients are classified based on the number of criteria met [35].

The ESAS questionnaire assesses nine common symptoms (pain, tiredness, nausea, depression, anxiety, drowsiness, appetite, well-being, and shortness of breath), with severity rated on a scale from 0 (absent) to 10 (worst possible severity) [29].

The PPS is a tool that evaluates the functional status of patients, including their ability to ambulate, perform self-care, and maintain oral intake, adjusted from the Karnofsky Performance Status scale [30]. All tools used in the study are validated in Italian.

The MBS is a vertical scale from 0 to 10, with numbers anchored to corresponding verbal expressions of progressively increasing intensity.

Study questionnaires and clinical assessment tools were administered by physicians or healthcare professionals, while the Edmonton Symptom Assessment Scale (ESAS) was completed directly by patients.

As an observational study, the objective was to evaluate the performance of NECPAL and PaP score in a real-world population of patients with advanced chronic diseases, without influencing clinical decision-making or referral pathways. Study assessments were performed independently of subsequent treatment decisions, and physicians remained free to continue routine clinical management according to local practice, including adoption of a general palliative approach or referral to specialist palliative care services when considered appropriate.

### Statistical analysis

Overall survival (OS) was defined as the time between the date of enrollment to the date of death for any cause or the date of last follow-up. OS was estimated using Kaplan-Meier method.

Quantitative variables were described as mean, standard deviation (SD) or median, and interquartile range (IQR) whereas categorical variables were described as frequency and/or percentage.

Sensitivity (proportion of individuals predicted to be deceased among those that did die), specificity (proportion of individuals predicted to be alive among those that were alive), positive predictive value (PPV) and negative predictive value (NPV) were calculated. The Receiver Operating Characteristic (ROC) curve and the area under the curve (AUC) were also calculated. The AUC was measured at 1, 3, 6, 9, and 12 months to assess the discriminative ability of the model in predicting the risk of death at different time points. The binomial proportion confidence intervals (95% CI) for these measures were computed using a normal approximation. No formal sample size calculation was performed. The study sample consisted of all consecutive eligible patients recruited during the predefined study period across participating centers; therefore, the sample size reflected the available study population rather than an a priori statistically determined sample size.

To minimize potential sources of bias, consecutive patients meeting the inclusion criteria were enrolled during the study period. Data collection was performed using standardized and validated assessment tools (NECPAL and PaP score) applied uniformly across all participants. Clinical and outcome data were prospectively recorded according to predefined variables, reducing the risk of information bias. Although the observational design does not allow control of all potential confounders, these methodological strategies were adopted to improve the reliability and consistency of the collected data.

Missing data were handled using complete-case analysis. Patients with missing data for a given instrument were excluded from analyses involving that instrument. The proportion of missing data was limited and is reported in the Results section where appropriate. No imputation methods were applied.

Regarding follow-up, survival status at 30 days and 12 months was ascertained for all included patients through clinical records and/or institutional databases. Therefore, no relevant loss to follow-up occurred for the primary outcome.

No sensitivity analyses were performed in this study.

Statistical analyses were performed with SAS software, version 9.4 (SAS Institute, Cary, NC, USA).

## Results

### Baseline characteristics of the study population

Between January 2021 and May 2025, 193 patients were enrolled: 102 with advanced cancer, 50 with chronic respiratory failure, and 41 with chronic renal failure.

Among patients with cancer, the median age was 71 years and 57% were male. Most (77%) had an ECOG performance status (PS) of 0–1. The most frequent diagnoses were non-small cell lung cancer (47.1%), pancreatic adenocarcinoma (30.4%), and gastric cancer (22.5%). 40% had multiple metastases and 8.8% had locally advanced disease (Table 1).

**Table 1:** Patient characteristics of the study populations

	Population A: cancer (*N* = 102)	Population B: pulmonary disease (*N* = 50)	Population C: renal disease (*N* = 41)	Total (*N* = 193)
	*N*. (%)	*N*. (%)	*N*. (%)	*N*. (%)
Age (years): median value (range, IQR)	71 (42–90, 65–77)	78 (59–89, 73–82)	80 (47–92, 74–84)	75 (42–92, 68–80)
Sex: Male	58 (56.9)	35 (70.0)	30 (73.2)	123 (63.7)
ECOG Performance Status				
0	35 (34.3)	1 (2.0)	0	36 (18.7)
1	44 (43.1)	16 (32.0)	14 (34.2)	74 (38.3)
2	19 (18.6)	23 (46.0)	8 (19.5)	50 (25.9)
≥3	4 (4.0)	10 (20.0)	19 (46.3)	33 (17.1)
Comorbidities	86 (84.3)	49 (98.0)	40 (97.6)	175 (90.7)
Type of comorbidites				
Cardiovascular	64 (62.7)	39 (78.0)	22 (53.7)	125 (64.8)
Diabetes mellitus	22 (21.5)	14 (28.0)	13 (31.7)	49 (25.4)
Gastrointestinal	19 (18.6)	16 (32.0)	5 (12.2)	40 (20.7)
Genitourinary	24 (23.5)	12 (24.0)	1 (2.4)	37 (19.2)
Solid tumors and hematologic malignancies	13 (12.7)	8 (16.0)	21 (51.2)	42 (21.8)
Endocrine	16 (15.7)	9 (18.0)	3 (7.3)	28 (14.5)
Respiratory	8 (7.8)	10 (20.0)	4 (9.8)	22 (11.4)
Psychiatric	9 (8.8)	7 (14.0)	2 (4.9)	18 (9.3)
Other	45 (44.1)	35 (70.0)	23 (56.1)	103 (53.4)
Charlson Comorbidity Index:				
median value (range, IQR)	4 (0–12, 3–5)	6 (2–14, 5–8)	10 (3–16, 7–12)	5 (0–16, 4–8)

*IQR* Interquartile range, *ECOG* Eastern Cooperative Oncology Group

In the pulmonary disease group, the median age was 78 years, 70% were male, and 34% had ECOG PS 0–1. Most patients had COPD (78%), while 22% had idiopathic pulmonary fibrosis (IPF). Dyspnea was moderate to severe in all patients, with 36% reporting very intense or unbearable breathlessness. Cough was generally mild (74%), though a minority experienced moderate or severe symptoms.

People living with renal disease had a median age of 80 years, 73% were male, and 34% had ECOG PS 0–1. Fifteen patients (36.6%) had end-stage renal disease, 5 (12.2%) vascular nephropathy, 3 (7.3%) glomerular disease, 4 (9.8%) urologic disease and 14 (34.2%) other heterogeneous conditions, including multiple myeloma, mantle cell lymphoma, AL or renal amyloidosis, monoclonal gammopathy/light chain deposition disease, and diabetes mellitus. Most were on dialysis (71%), mainly hemodialysis (93%).

Comorbidities were highly prevalent, particularly in non-oncological groups, with cardiovascular and metabolic diseases being most common.

### Questionnaire and assessment tools

All 193 patients completed the study assessments except for the PaP score, which was unavailable in 14 patients because of missing recent blood tests. The average number of questionnaires completed per patient was 6.

At baseline, the PaP Score classified most patients into risk group A, with a 30-day survival probability greater than 70% (99% of patients with cancer, 93% of people living with pulmonary disease, and 80% of people living with renal disease). The remaining patients were in risk groups B and C, with lower survival probabilities (Table 2).

**Table 2:** Questionnaires evaluated at baseline

	Population A: cancer	Population B: pulmonary disease	Population C: renal disease	Total
	No. (%)	No. (%)	No. (%)	No. (%)
PaP score				
Risk group A: 30-day OS > 70%	95 (99.0)	39 (92.9)	33 (80.5)	167 893.3)
Risk group B-C: 30-day OS ≤ 70%	1 (1.0)	3 (7.1)	8 (19.5)	12 (6.7)
NECPAL				
NECPAL-: SQ-	39 (38.6)	25 (50.0)	3 (7.3)	67 (34.9)
NECPAL-: SQ + and 0 criteria	2 (2.0)	4 (8.0)	0	6 (3.1)
NECPAL+: SQ + and 1–2 criteria	34 (33.7)	7 (14.0)	1 (2.5)	42 (21.9)
NECPAL+: SQ + and > 2 criteria	26 (25.7)	14 (28.0)	37 (90.2)	77 (40.1)
PPS				
100 − 90	52 (51.0)	4 (8.0)	7 (17.1)	63 (32.6)
80 − 70	43 (42.1)	28 (56.0)	14 (34.1)	85 (44.1)
60 − 50	5 (4.9)	18 (36.0)	12 (29.3)	35 (18.1)
40 − 0	2 (2.0)	0	8 (19.5)	10 (5.2)

*PaP* Palliative prognostic, *NECPAL* Necesidades paliatives, *OS* Overall survival, *SQ* Surprise question, *PPS* Palliative Performance Scale

Regarding the NECPAL tool, 61.7% of the population was classified as NECPAL positive (SQ + and at least one positive criterion). Most patients with cancer (59.4%) and people living with renal disease (92.7%) were NECPAL positive, while more than half of the people living with pulmonary disease (58%) was NECPAL negative.

### Symptom burden and functional status

**Fig. 1:**
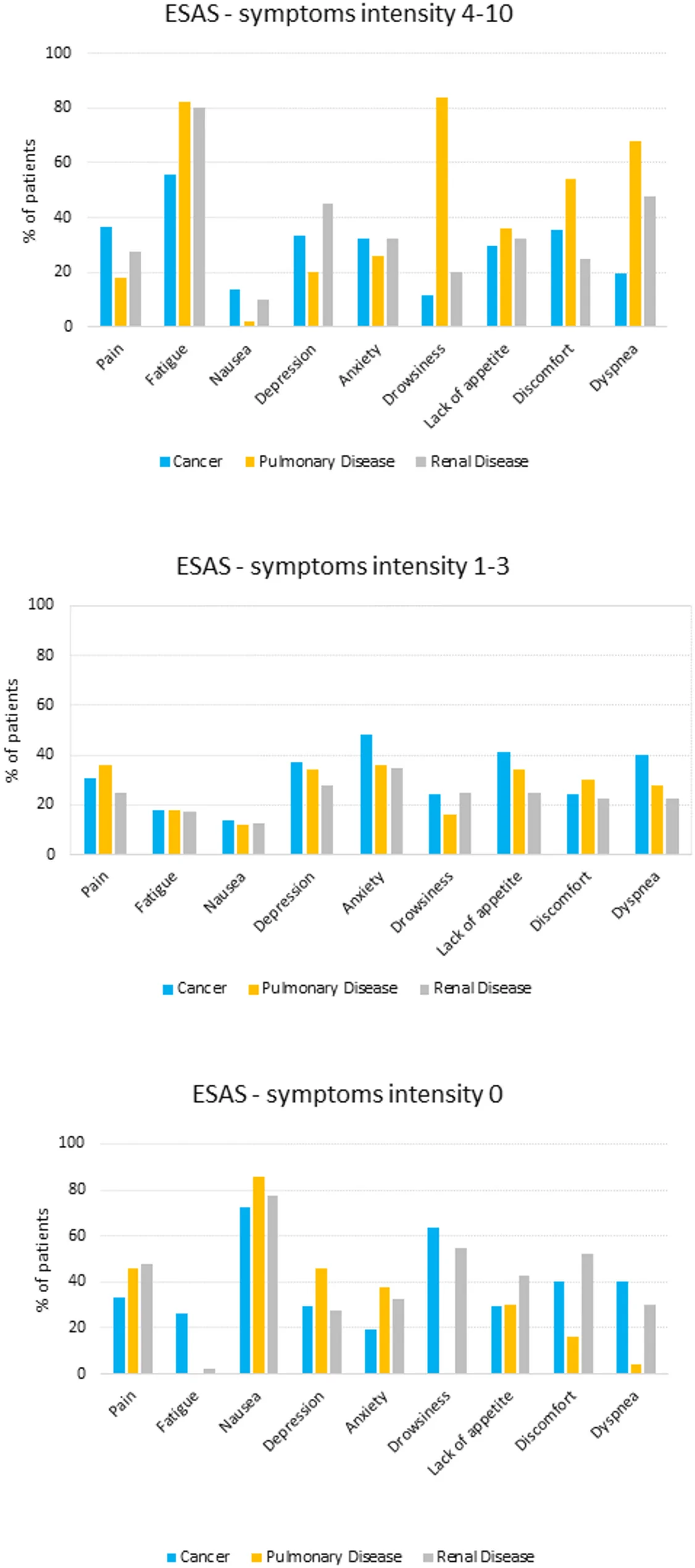
Edmonton Symptoms Assessment System (ESAS) intensity at baseline

Symptom prevalence and intensity differed across disease groups (Fig. 1). Fatigue (ESAS ≥ 4) was highly prevalent in people living with pulmonary disease (82%) and renal disease (80%), compared with patients with cancer (55.9%). Dyspnea was most intense in people living with pulmonary disease (68%), whereas pain was more prevalent in patients with cancer (36.3%).

Functional status also varied across groups. Patients with cancer showed the best performance status, with 51% having a PPS of 90–100%. In people living with pulmonary disease, 56% had a PPS of 70–80%, while 34.1% of people living with renal disease had a PPS of 70–80%.

Overall, symptom burden and functional status varied according to disease category, with different patterns observed across oncological and non-oncological conditions.

### Performance characteristics of PaP score and NECPAL over time

**Fig. 2:**
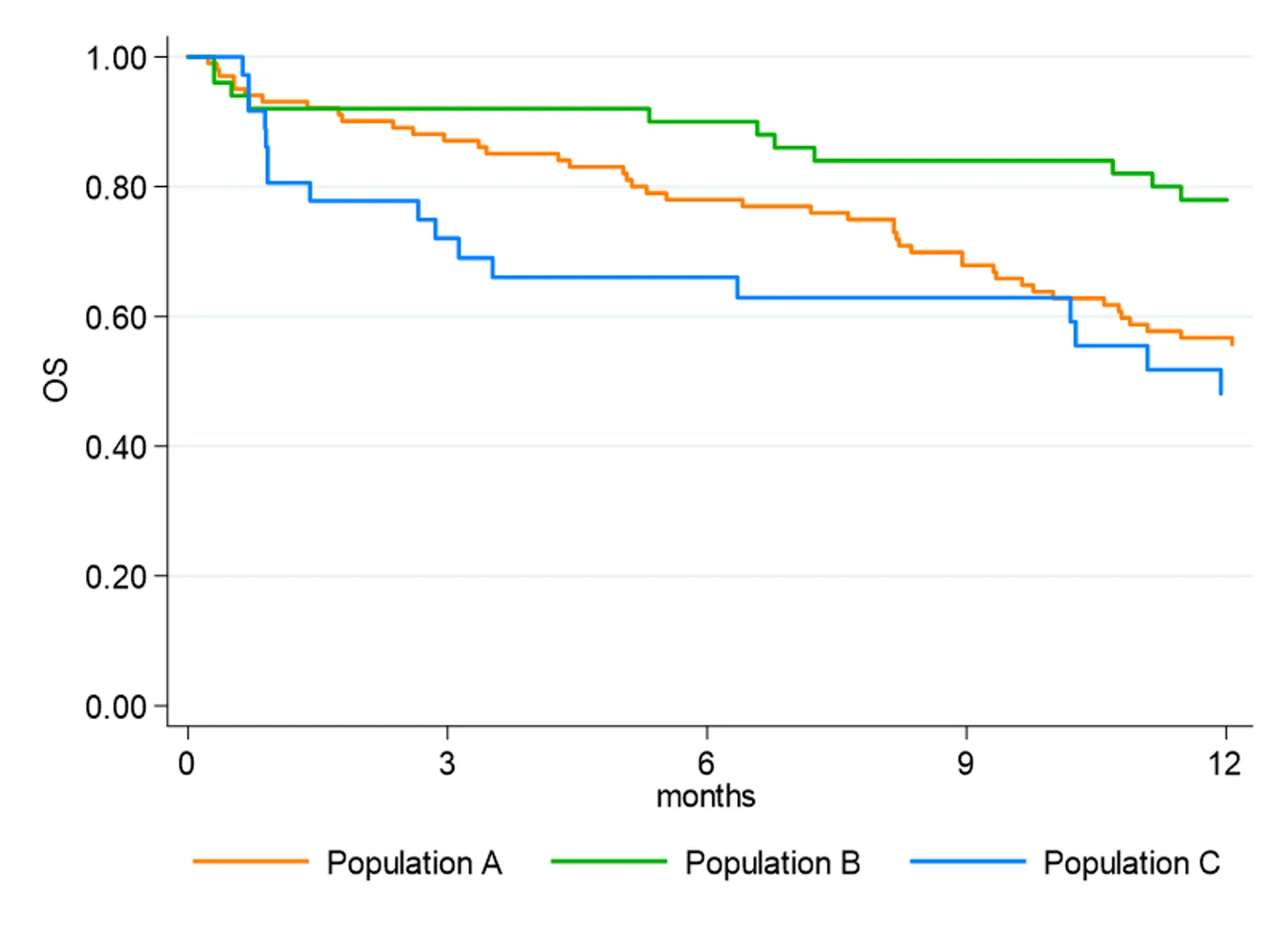
Kaplan-Meier overall survival of the study populations

The overall survival at 1 month was 93% (95% CI 88–98) for the patients with cancer, 90% (95% CI 82–98) for the people living with pulmonary disease, and 89% (95% CI 79–99) for the people living with renal disease. At 12 months, the overall survival was 57% (95% CI 47–100) for patients with cancer, 78% (95% CI 78–97) for people living with pulmonary disease and 57% (95% CI 40–74) for people living with renal disease (Fig. 2).

Table 3 reports the sensitivity, specificity, positive and negative predictive value of both PaP score and NECPAL tools at 1 and 12 months. At 1 month, PaP score presents high sensitivity (100%) and specificity (87.7%, 95% CI 82.5–92.9) and low PPV (29.6%, 95% CI 12.4–46.8) whereas NECPAL presents high sensitivity (89%, 95% CI 81–95) but low specificity (38.8%, 95% CI 31.4–46.2) (explained by high number of false positives) and PPV (4.7%, 95% CI 0.7–8.7) (explained by low number of true positives and high number of false positives).

**Table 3:** Sensitivity, specificity, positive and negative predictive value of PaP score and NECPAL at 1 and 12 months

	PaP score (cutoff 5)	NECPAL (-/+)	PaP score (cutoff 5)	NECPAL (-/+)
	1 month		12 months	
Sensitivity % (95% CI)	100 (-)	89.0 (81.0–95.0)	20.0 (0.1–44.8)	87.5 (64.6–100)
Specificity % (95% CI)	87.7 (82.5–92.9)	38.8 (31.4–46.2)	88.9 (83.6–94.2)	41.2 (33.3–49.1)
PPV % (95% CI)	29.6 (12.4–46.8)	4.7 (0.7–8.7)	11.8 (0.1–27.1)	7.5 (2.1–12.7)
NPV % (95% CI)	100 (-)	100 (-)	93.7 (89.5–97.9)	98.4 (95.3–100)

*PPV* Positive predictive value, *NPV* Negative predictive value

**Fig. 3:**
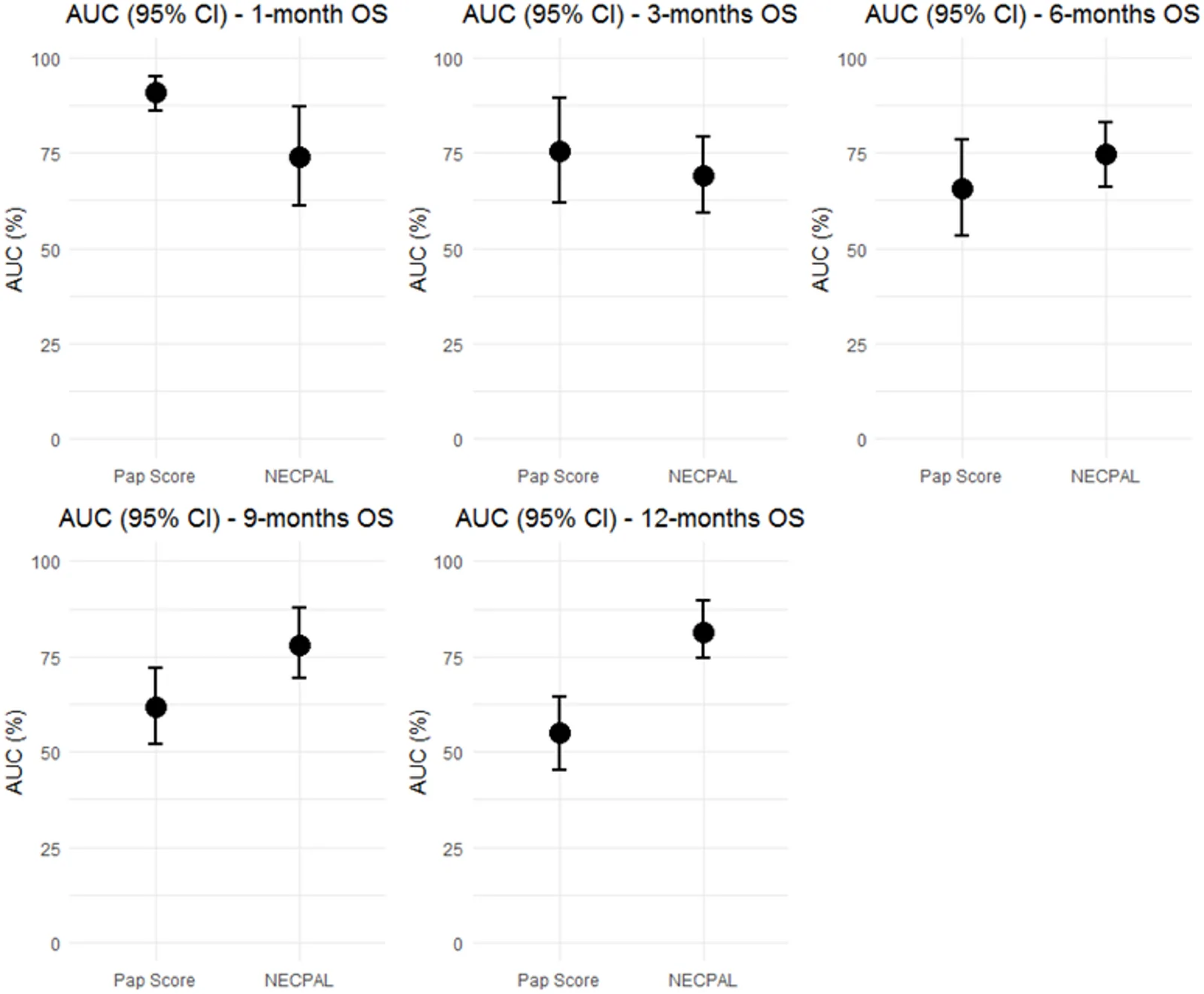
Area under the ROC curve for the PaP score and NECPAL over time

At 12 months, NECPAL improves specificity (41.2%, 95% CI 33.3–49.1), with a sensitivity of 87.5% (95% CI 64.6–100) whereas PAP score presents a specificity of 88.9% (95% CI 83.6–94.2) but a low sensitivity (20%, 95% CI 0.1–44.8). Similar results were seen in the 3 study populations. Regarding the discriminative ability of PaP score and NECPAL, the AUC of the PaP score was progressively decreasing through time, reporting a good discrimination at 1 and 3 months (91%, 95% CI 86–95; 76%, 95% CI 62–89, respectively). The model failed to discriminate between individuals with a risk to die or to be alive at 6, 9 and 12 months, with AUC below 65% at 9 and 12 months (Fig. 3). Regarding NECPAL, the AUC was higher than that of the PaP score starting from the sixth month: 75% (95% CI 66–83) at 6 months, 78% (95% CI 69–88) at 9 months and 81% (95% CI 75–89) at 12 months.

### Healthcare resource utilization

Regarding healthcare resource utilization, hospitalization in acute care units was more frequent in the patients with cancer compared to the other populations, and the cumulative days of hospitalization were higher for the people living with pulmonary disease. 33% of the people living with pulmonary disease required ICU admission during hospitalization, with a median ICU stay of 12 days. Additionally, 70% of people living with pulmonary disease had at least one access to the emergency room (Table 4).

**Table 4:** Healthcare resource utilization

	Population A: Cancer		Population B: Pulmonary disease	Population C: Renal disease
	No. (%)		No. (%)	No. (%)
Hospitalization in acute units	56 (70.9)		24 (48.0)	15 (46.9)
Number of hospitalization in acute units				
1	31 (55.4)		11 (45.8)	9 (60.0)
2	11 (19.6)		4 (16.7)	4 (26.7)
≥3	14 (25.0)		9 (37.5)	2 (13.3)
Cumulative days of hospitalization in acute units: median value (range, IQR)	16 (1–89, 10–32)		29 (2–151, 10–45)	20 (1–150, 7–48)
Need for ICU during hospitalization	4 (7.1)		8 (33.3)	4 (26.7)
Number of days of ICU stay: median value (range, IQR)	8 (2–13, 4–11)		12 (5–31,6–18)	11 (5–50, 7–31)
Access to emergency room	52 (65.8)		35 (70.0)	8 (25.0)
Number of access to emergency room: median value (range, IQR)	2 (1–6, 1–3)		1 (1–8,1–5)	1 (1–2, 1–2)
Palliative care setting use				
Palliative care outpatient clinic	33 (41.8)	11 (22.0)		1 (3.1)
Number of palliative care outpatient visit: median value range (range, IRQ)	2 (1–10, 1–5)	1 (1–7, 1–2)		1 (1–1, 1–1)
Hospice admission	24 (30.4)	1 (2.0)		2 (6.2)
Home palliative care program	28 (35.4)	8 (16.0)		3 (9.4)
Cumulative number of days within palliative care program: median value (range, IQR)	29 (4–275, 11–83)	162 (5–475, 28–383)		21 (0–205, 0–205)

*IQR* Interquartile range, *ICU* Intensive care unit

Palliative care utilization was most frequent in patients with cancer: 42% attended a palliative care outpatient clinic, 30% were admitted to hospice, and 35% participated in a home palliative care program. The home palliative care program was used by 16% of the people living with pulmonary disease, with a median cumulative duration of 162 days in the program (Table 4).

Disease progression was the leading cause of death in both patients with cancer (72.3%) and people living with renal disease (61.9%), with death in a palliative care setting (home + hospice) being more common in the patients with cancer (50.8%) compared to the people living with renal disease (29.5%) and people living with pulmonary disease (10%).

## Discussion

In this study, we explored the prognostic performance of the NECPAL instrument [20, 24], originally developed to identify palliative care needs, and the PaP score [21–23] at several time points in three cohorts of people living with advanced chronic diseases. Our findings suggest that PaP score and NECPAL provide different types of clinical information. PaP score demonstrated better short-term prognostic performance, whereas NECPAL, although primarily designed to identify palliative care needs, showed greater association with longer-term survival outcomes.

At 30 days, the AUC was 91% for the PaP score and 74% for NECPAL, while at 12 months the AUC increased to 81% for NECPAL and decreased to 54.8% for the PaP score. These findings suggest that the two instruments may be used for different clinical purposes and time horizons. Our results are partially consistent with those reported by Esteban-Burgos et al. [36], who evaluated NECPAL v4.0 in older nursing home residents with advanced chronic conditions. Unlike earlier versions, NECPAL v4.0 incorporates prognostic components that may improve predictive performance. In their study, NECPAL showed greater prognostic accuracy at 12 months (58.9%) compared with other screening tools, whereas prognostic accuracy at 3 months was lower. Although direct comparison with our findings should therefore be interpreted cautiously, both studies support the usefulness of NECPAL in identifying people living with advanced chronic non-oncological diseases who may benefit from palliative care assessment.

Moreover, sensitivity, specificity, and NPV were 87.5%, 41.2%, and 98.4% for NECPAL, and 20%, 88.9%, and 93.7% for the PaP score at 12 months. Gómez-Batiste X et al. [20] report similar results for the NECPAL tool, with a sensitivity of 91.3%, specificity of 32.9%, and NPV of 91% at 12 months. However, while Gómez-Batiste X et al. show a PPV of 33.5% at 12 months for NECPAL, this index was lower in our study, both for NECPAL (7.5%) and for the PaP score (11.8%). This difference may reflect the heterogeneity of our population, the relatively limited sample size, and the inclusion of patients at earlier stages of disease progression.

The differing prognostic trajectories of oncological and non-oncological diseases may also explain the distinct performance of the two tools [37–40]. Patients with advanced cancer often experience a relatively predictable decline near the end of life, whereas people living with advanced chronic non-oncological diseases frequently show slower and more heterogeneous trajectories, characterized by periods of stability interrupted by acute exacerbations [41]. This variability makes prognostic estimation particularly challenging in non-oncological populations and may contribute to the lower short-term prognostic accuracy observed with NECPAL.

In our cohort, people living with pulmonary and renal diseases showed greater functional dependence and multimorbidity burden compared with patients with oncological diseases. These findings may suggest that referral criteria for palliative care in non-oncological populations remain more restrictive in clinical practice, despite the substantial symptom burden experienced by these individuals. As observed in our symptom analysis, symptom intensity in people living with advanced chronic non-oncological diseases may be comparable to that observed in patients with cancer, further supporting the need for systematic assessment of supportive and palliative care needs across disease categories.

The evidence supporting palliative care interventions in chronic non-cancer illnesses has progressively increased. Quinn et al. [42], in a systematic review and meta-analysis, demonstrated that palliative care interventions in adults with chronic non-cancer illness were associated with improvements in quality of life and symptom burden. Similarly, Temel et al. [9] demonstrated the benefits of early integrated palliative care in patients with metastatic non-small cell lung cancer. However, evidence from oncology populations should not be directly generalized to non-oncological diseases because of substantial differences in disease trajectories, prognostic uncertainty, and models of care delivery [37, 38].

In Italy, the statement of the Società Italiana di Anesthesia, Analgesia, Rianimazione e Terapia Intensiva (SIAARTI) bioethics research group [28] identified patients with non-oncological diseases for whom transition to palliative care may be appropriate, partially adopting the Gold Standards Framework Proactive Identification Guidance strategy [39]. Nevertheless, palliative care and intensive care are still frequently considered alternative approaches rather than potentially integrated models of care [28].

A substantial proportion of individuals in the renal disease subgroup had previous solid or hematologic malignancies. However, this subgroup was clinically heterogeneous, including different oncologic conditions with limited representation of primary hematologic malignancies. Therefore, although these comorbidities may have influenced prognosis, their overall impact on the study findings is likely limited.

Our study has shown the varying use of health resources across different populations. Palliative care services have proven their ability to impact the quality of life of individual patients [43], as well as the quality of care in terms of lower utilization of intensive care units, reduced emergency admissions and readmissions, lower hospital death rates, and cost savings [44–48]. Moreover, early palliative care facilitates earlier referral to end-of-life palliative care and increases the likelihood of death in a palliative care setting [49–51].

The strengths of this study lie in the diversity of medical conditions and the concurrent evaluation of NECPAL and PaP score within the same multicenter cohort, which has not been used before. The present study does not allow conclusions regarding the effectiveness of a combined strategy. Nevertheless, the observed differences in prognostic performance suggest that future studies could explore whether integrating information from both instruments may improve clinical decision-making. These tools are adaptable to various healthcare and social care environments. Future studies could evaluate whether information derived from these tools may support the development and implementation of comprehensive programs that integrate early identification of palliative care needs with appropriate interventions, such as needs assessment, advanced care planning, and case management, within an integrated, population-based, community-oriented care model. If confirmed in future studies, such approaches could contribute to more structured pathways for identifying patients with palliative care needs and supporting care planning within chronic disease management programs.

This study has several limitations. First, although multicentric, the study included a relatively limited sample size. Second, the observational design limits causal interpretation of the findings. Third, comparisons with previous studies should be interpreted cautiously because of differences in patient populations and versions of prognostic tools used, particularly regarding NECPAL v4.0.

The multicentre design and the heterogeneity of the included populations may support the external validity and applicability of the findings across similar real-world clinical settings. Nevertheless, differences in healthcare organization, palliative care pathways, and referral practices across institutions and countries may influence the transferability of the results. These findings should be interpreted as descriptive contextual data and were not intended to evaluate the effect of either instrument on healthcare utilization. Moreover, the predictive performance of both instruments showed relevant limitations, particularly regarding positive predictive value and specificity for NECPAL, and sensitivity for PaP at longer follow-up. Despite these limitations, this study provides clinically relevant information regarding the prognostic role of the PaP score and NECPAL in people living with advanced chronic diseases. The PaP score showed greater accuracy for short-term prognosis and although NECPAL was not initially developed as a prognostic instrument, it showed better association with longer-term survival outcomes in this cohort. The two instruments provide different types of information regarding prognostic assessment and identification of palliative care needs; however, whether integrating information from both instruments improves clinical decision-making remains to be established. Finally, future studies may explore whether artificial intelligence and machine learning approaches [52] can complement traditional prognostic instruments.

## Conclusion

In this multicenter cohort of patients with advanced chronic diseases, the PaP score showed better performance for short-term survival prediction, whereas NECPAL, a tool primarily designed to identify palliative care needs, showed greater association with longer-term survival outcomes. Both tools showed important limitations in predictive accuracy, particularly the low specificity and PPV observed for NECPAL and the reduced long-term sensitivity of the PaP score. Given the limited predictive performance observed for several measures and the observational design of the study, these findings should be interpreted cautiously. Further studies are needed to determine whether integrating information from both instruments improves clinical decision-making.

## Supplementary Information

Supplementary Material 1.

## Data Availability

The datasets generated and/or analyzed during the current study are available from the corresponding author upon reasonable request.
